# Gender stereotypes and education: A comparative content analysis of Malaysian, Indonesian, Pakistani and Bangladeshi school textbooks

**DOI:** 10.1371/journal.pone.0190807

**Published:** 2018-01-19

**Authors:** Kazi Md. Mukitul Islam, M. Niaz Asadullah

**Affiliations:** 1 Faculty of Economics and Administration, University of Malaya, Kuala Lumpur, Malaysia; 2 Political and Press Section, German Embassy, Dhaka, Bangladesh; 3 Department of Economics, University of Reading, Reading, United Kingdom; 4 Centre on Skills, Knowledge and Organisational Performance (SKOPE), Department of Education, University of Oxford, Oxford, United Kingdom; 5 School of Education, Environment and Development, University of Manchester, Manchester, United Kingdom; 6 IZA Institute of Labor Economics, Bonn, Germany; Universitat Jaume I, SPAIN

## Abstract

Using government secondary school English language textbooks from Malaysia, Indonesia, Pakistan and Bangladesh, we conducted a quantitative content analysis in order to identify gender stereotypes in school education. In total, 21 categories of exclusion and quality of representation were used to study gender stereotypes. Our analysis confirms a pro-male bias in textbooks: the aggregate female share is 40.4% in textual and pictorial indicators combined. Female occupations are mostly traditional and less prestigious while the characters are predominantly introverted and passive in terms of personality traits. Women are also shown to be mostly involved in domestic and in-door activities while men have a higher presence in professional roles. Systematic underrepresentation of females is evident regardless of whether we look at the text or pictures. A cross-country analysis shows that the female share in picture content is only 35.2% in Malaysia and Bangladesh. Overall, the proportion of female to male characters (text and pictures combined) is balanced in Malaysia and Indonesia (44.4% and 44.1% respectively) while this share is only 24.4% and 37.3% in Pakistani and Bangladeshi textbooks respectively. The finding of underrepresentation of women in Pakistani textbooks, in terms of quality and quantity, is robust to the selection of province-, grade- and subject-specific textbooks, as well as the range and type of categories used.

## Introduction

Despite their growing presence in the labor force and educational institutions in the last few decades, women remain socially marginalized and underrepresented within, as well as, outside of households in low-income countries [1,2]. More than 700 million women worldwide are married off each year before they reach their 18^th^ birthday while one in every three women experience physical violence by their intimate partners [3]. Women are also paid significantly lower wages than men [4]. They also account for almost two-thirds of the 775 million illiterate population today [5]. A contributing factor to the lack of improvement in women’s socioeconomic status is the persistent male-female gap in educational opportunities as well as in social and gender norms, which interfere with the ability of girls and women to take advantage of the opportunities in many developing countries [6–8].

Investment in women’s education is widely believed to benefit the society through numerous economic and noneconomic channels [9–17] [114]. Given the evidence of the private and social returns to female education and various international agreements and national campaigns, it is not surprising that gender inequality in school enrolment has reduced in many developing countries in the last few decades. In developing countries, the average years of female schooling have increased from 2.2 years to 7.2 years between 1970 and 2009 [18]. Between 2006 and 2013, female net enrollment in secondary education increased from 57% to 65% [19].

Schools serve as the dominant institution for transmitting social knowledge and attitudes, thereby facilitating social change [20]. Therefore, some consider higher school enrolment of girls to reduce gender inequality in society, using a broad range of indicators, by shifting social attitudes in favor of women [8, 21]. In reality, however, educational institutions are not designed or mandated to shift social attitudes in favor of women. Exposure to institutional education may not be enough to alter gender attitudes and address gender stereotypes. According to some scholars, the classroom can paradoxically serve as a place for nurturing gender bias and stereotypes [6] [22–24]. For instance, in Africa, it is often the maxim in the classroom that teachers say “boys need career and girls need husbands” [23]. School education can even be a negative experience. For instance, religious schools often regulate the socialization process in ways that particularly disadvantage girls [25]. Moreover, there are pro-male attitudes among teachers [26] and gender imbalance in the teaching staff. Lastly, schools may rely on overly masculine textbooks for girls’ education [26, 27]. According to Blumberg [6], gender bias in the textbook is one of the “hardest budge rocks in the road to gender equality in education” and is geographically more widespread than the gender gap in school enrolment. The United Nation’s Girl Education Initiative identified it as one of the five challenges towards achieving gender equality in education [28]. Biased textbook contents not only limit women’s worldviews and career choices, but they also distort their self-image and the image of the opposite gender group [29]. Yet, compared to other school-specific drivers of gender inequality, textbook content is less researched and frequently overlooked in the policy debate. This is a serious concern given the evidence that students spend the majority (80%-95%) of their classroom time using textbooks [30]. Most teachers also rely on textbooks for assigning homework for the students [31]. Research on classroom practices from developing countries shows that teachers barely challenge the textbook stereotypes and instead reproduce them; this only propagates the problem, while students passively receive what they are taught [32].

Our study, therefore, examines the presence of gender stereotypes in school textbooks in four countries in South and South-East Asia, namely, Malaysia, Indonesia, Pakistan and Bangladesh. These countries were chosen because they belong to different levels of socio-economic development and vary significantly in terms of progress in female schooling despite their patriarchal social structure. The sample countries also have a predominantly Muslim population, so an analysis of these countries can shed additional light on the relatively higher gender inequality in Muslim countries in education and social indicators [11,33]. To the best of our knowledge, this is the first comparative study on gender stereotypes in school textbooks of these four major Muslim countries.

Our research objectives are to document the extent of (a) gender exclusion and improper representation in the texts and images of the textbooks and (b) the variation in gender stereotypes across countries. In terms of study population, we exclusively focus on government-approved English textbooks used in secondary schools (grade 9). The content analysis technique is applied to examine gender imbalances in textbooks employing as many as 21 indicators. Since our main analysis is based on a single textbook from each of the four countries, we further examine ten additional textbooks from Pakistan to assess the sensitivity of our findings.

The rest of the paper is organized as follows. Section 2 provides an overview of the sample countries regarding aggregate indicators of economic performance, women’s economic and political participation, educational and demographic achievements and constitutional articles/rights. Section 3 presents an extensive review of the literature on textbook content analysis. Section 4 discusses the methodology and data. Section 5 presents the main results while Section 6 concludes.

## Study background: Women’s schooling and socioeconomic status in the sample countries

Women in South and South-East Asia face a multitude of social and economic problems. Their participation in the labor market is not only low, but they are also less likely to be employed compared to men (see Table 1). On average, only 55 females participate in the labor force for every 100 males in our sample countries. Pakistani women are particularly disadvantaged regarding labor market indicators and contraceptive prevalence rate, where women also face a greater burden of housework owing to larger family size.

**Table 1:** Women’s development indicators and policy provisions.

Indicators	Malaysia	Indonesia	Pakistan	Bangladesh
**Economic Performance and Participation**				
GNI per capita, (current US$) ^a^	10,760	3,630	1,410	1,080
Female-male labor participation ratio (ILO estimate) ^a^	0.59	0.62	0.30	0.70
Female-male employer ratio (for every 100 males) ^a^	11.1	20.5	2.1	12.8
Employment to population ratio, 15+, female (%) ^a^	42.9	47.5	22.2	54.5
Wage difference (female wage as % of male) ^c^	58.0	49.0	19.0	52.0
**Human and Gender Development Rankings**				
Gender Related Development Index (GDI) ^b^	91	98	145	107
Global Gender Gap ranking ^c^	111	92	144	64
Human Development Index (HDI) ^b^	62	110	147	142
**Educational Development**				
Literacy rate, adult female (%, ages 15 and older) ^a^	90.7	93.5	44.2	69.9
Literacy rate, youth female (%, between age 15–24) ^a^	98.4	99.6	65.5	93.5
Secondary school enrollment (total gross) ^a^	70.0	83.0	38.0	53.0
Secondary school enrollment (girls to boys ratio) ^a^	0.94	0.98	0.73	1.12
Female teachers in secondary school, % ^f^	65.3	53.1	57.1	20.8
**Demographic Status**				
Total fertility rate (births per woman)^a^	2.1	2.3	3.5	2.1
Contraceptive prevalence rate, any method (% woman ages 15–49) ^a^	49.0	63.0	35.0	62.0
**Political Empowerment**				
Women in parliament (%) ^d^	10.4	18.6	20.7	20.0
Women in ministerial positions (%) ^d^	6.0	23.0	0.0	7.0
**Constitutional Articles/Rights**				
Equality and non-discrimination ^e^	8,135	28I	Preamble, 25	28
Public authority, institution and services ^e^	12	31	37	19
Education ^e^	12	28D	38	17
Political participation ^e^	12	28D	32,34 (60 reserved seat)	38 (50 reserved seat)
Employment ^e^	8	28D	27	29
Equal before law ^e^	8	27	25	27

Source

^a^ The World Bank Development Indicators

^b^ Human Development Report 2015

^c^ Global Gender Gap report 2015

^d^ UN Women 2016

^e^ Constitutional Database, UN Women 2017

^f^ UNESCO 2016.

Note: All figures are at national (country) level.

However, there are notable differences between South and South-East Asia (see Table 1 e.g. [1,19,41–44]). In Bangladesh and Pakistan, female literacy rates and gross female enrolment are almost half that of Malaysia and Indonesia. The under-investment in girls’ education continues despite the fact that female education has many non-economic direct and indirect benefits [12] [34–38]. Schooling also matters significantly for the labor market performance of women in South and South-East Asia [39,40]. Solely owing to reservation policies, Pakistan and Bangladesh have more female parliamentarians.

In recent decades, governments in these four countries have responded to the existing gender gaps in social and labor market indicators by enacting new laws that aim to improve women’s access to education as well as job opportunities. In Bangladesh and Pakistan, financial incentives have been provided to girls for continuing on to secondary school and/or delaying marriage [17,45,46]. Constitutions in the four countries studied promise equality, non-discrimination, the right to education and political participation for women (See Table 1). All countries have National Women Policies that emphasize equal rights for women in the socio-economic domain. At the same time, patriarchal mindsets often demand reforms that undermine pro-women policies. Examples include virginity tests for female police recruitment in Indonesia [47], the reform of a marriage law in Bangladesh and Malaysia allowing for child marriage under “exceptional circumstances” [17, 46, 48], a discriminatory application of family law denouncing Malaysian women as second class citizens [49] and the practice of honor killings in Pakistan [50].

Therefore, women remain poorly represented in economic as well as political spheres (e.g., the share in ministerial positions). The rising enrolment of females in schools (primary, secondary or even higher) per se does not guarantee gender empowerment or equality. Patriarchal customs and social norms continue to affect gender roles through socialization processes at school and at home. This, in turn, shapes women’s attitudes towards further education and the job market [51]. Women in the region face many social barriers outside the education system and the schooling of young women can enhance their voice and power within the family [50]. However, a gender insensitive school curriculum may particularly undermine this important role of education in addressing the existing sources of gender inequality in society. Universal access to education can, paradoxically, serve as an institutional source of discrimination through the teaching system, classroom environment and the school curriculum [23]. This is an important concern because all countries have agreed to the Sustainable Development Goals (SDGs) and joined the global community in making ambitious commitments to gender equality in education. Thus, they are bound to the indicative strategy recommended in Education 2030 Target 4.5 of SDG 4, namely, “Ensure government review of education sector plans, budgets, curricula and textbooks, along with teacher training and supervision, so that they are free of gender stereotypes and promote equality, non-discrimination and human rights and foster intercultural education”[52]. The next section elaborates on this concern by critically reviewing the theoretical and empirical literature on the role of textbooks in gender development.

## Literature review

In this section, we first focus on the theories of gender stereotypes based on differences in school curricula only. In the second part of this section, we present an overview of the existing studies on textbook content analyses with a particular focus on developing country studies.

There are two theories of gender stereotypes within an educational institution, namely, social cognitive theory and hidden curriculum theory. The *social cognitive theory* emphasizes three types of environmental structures in schools: imposed, selected and constructed. In imposed environmental settings, teachers, curricula, textbook content and the class environment are all forced upon a child regardless of his/her personal preference [53]. In such a setting, they develop gender perceptions based on what they learn through the curriculum, teachers and other mechanisms at school. The *hidden curriculum theory* helps us to understand how gender stereotyped attitudes are reproduced in an imposed environment in schools. It maintains that school curriculum teaches something beyond the scope of the existing (formal) curriculum which is often implied and delivered through textbooks, teachers or other instruments [54]. As such, textbooks, staffing patterns, and the system of rewards, all contribute to promoting gender stereotypes at school, lowering self-esteem and undermining aspiration among for girls [23].

Jones, Kitetu, and Sunderland illustrate the interplay between hidden curriculum and its implication for cognitive and pedagogical development using gender imbalance in textbook ‘dialogues’[55]. First, if examples of dialogue between individuals of a particular gender are less frequent in the book, then students of the silenced gender will have fewer opportunities as dialogue participants. Second, if one sex initiates conversation most of the time, then the other sex will have inactive/passive participation in the dialogue. Finally, the negative cognitive impact of this is that female students consciously or unconsciously become demotivated to play roles that are restricted linguistically as well as occupationally. Therefore, this marginalization within textbooks (i.e. biased gender modeling through hidden curriculum) indirectly shapes female students’ acceptance of disempowered roles.

With regards to hidden curricula, there are two reasons why education materials succeed in instilling values and attitudes in the minds of young students. First, students accept what is imposed on them and are less critical about it. Second, most of their learning time is spent reading textbooks and paying attention to the messages conveyed through these books [56]. Moreover, in Asian countries, textbooks play an important role in the education system—the common perception is that whatever is printed in the textbook must be practiced [57,58].

Motivated by the social cognitive and hidden curriculum theories of gender stereotypes, numerous studies have examined teaching and learning materials to analyze the extent of the problem. The content analysis technique is one of the most common approaches to studying gender stereotypes in school textbooks in both developing countries (e.g., [6],[27] [59–62]) and developed countries (e.g.,[6] [62–66]). The simplest method involves coding the text and counting concepts, words and occurrences and reporting them in tables [67]. The key steps involve selectingthe sample text (whole book or specific chapters), identifying the unit of analysis (e.g. words, sentences, etc.), developing categories, reviewing the text to categorize it, counting and logging the occurrences of categories and statistical analysis facilitating interpretation [68]. Cohen described the process in 4Cs: coding, categorizing, comparing and concluding. Different scholars have suggested detailed and systematic steps to conduct content analysis research [60,62]. In this study, we have followed 11 steps detailed by Cohen [67,69].

One of the benefits of choosing content analysis is that the data is in a permanent form (texts and pictures) and verifiable as well as replicable through repeated analysis [67]. The literature using the content analysis method to explore gender bias has grown in developing countries in the past two decades [6,62]. Some of these studies only focus on picture-based analysis while others additionally scrutinize textual content. The latter group also varies regarding the extent to which they study exclusion and the quality of representation. Studies also differ regarding the selection of grade- and subject-specific textbooks.

Among early studies in Muslim developing countries, textbooks in Syria were found to portray males as engaging in a bustling world, while females were in the background in servitude, often degraded and victimized [27]. Similarly, in Nigeria, an analysis of 15 of the most frequently taught novels from 15 English literature textbooks found that all the stories were male-centered; out of 273 characters, only 61 were female [70]. A comparative analysis of gender stereotypes in the textbooks of two Arab countries found that textbooks in Jordan featured female characters 20.8 percent of the time as compared to almost zero or no mention of achievements of women in Palestine [71]. Likewise in Iran, some studies pointed out female exclusion and the quality of representation in the textbooks (e.g., [61] [72–74]). Bahman and Rahimi, for instance, examined different aspects of gender bias in 3 volumes of English textbooks taught in secondary schools in Iran [72] and found 70%-80% names, nouns, pronouns, and adjectives to be male specific.

Research in non-Muslim developing countries also suggests similar patterns. Studies in India show a high rate of male chauvinism in the textbook contents [75–77]. Women are found in less than one tenth of the textbooks in the Andhra Pradesh region [75] and around one fourth in the Rajasthan region [77]. Similar to India, studies in African countries show relative underrepresentation as well as poor quality of representation of females. In Zambia, for instance, the content analysis of secondary school textbooks found that 73.9% of the examples involved males shown as hard working with wage employment [78]. In China, primary school textbooks were found to over-represent males in all categories, the most stereotyped of which were professional occupations [79].

For our sample countries, existing content analysis studies describe women in passive and derogatory roles and limit their involvement to indoor activities [59, 80, 81]. One Malaysian study found 66% of all verbs used in the textbook to be male-specific [80]. Another study finds that textbooks function as channels for the indoctrination of sexism among young Malaysians [59].

In Indonesia, women were not only underrepresented in the English textbooks of higher grades in secondary school, but men were given a wider range of roles (62.4% as opposed to 37.2% in the case of women) [82]. The presence of gender stereotypes was also confirmed using a 12^th^ grade English textbook in Indonesia where the authors identified 1,098 (77%) male characters in comparison to only 321 (23%) female characters [83].

In Pakistan, Zeenatunissa examined 7 English and Urdu language textbooks at the secondary level [60]. She found that in only about 15% to 20% cases women were portrayed as leading characters, regular characters and subjects of biographies. Meanwhile, of the 50 occupations mentioned in the textbooks, women were only assigned to 8. Their activities were service-oriented, whereas men were in power-oriented activities. Mirza conducted by far the most comprehensive study on textbook contents in Pakistan [84]. It included as many as 194 textbooks from 4 provinces, spanning grades 1 to 10. Her study found only 26.5% of women as central characters. Regarding professional characters, only 15% were female in primary level textbooks and 9.8% in secondary level books. Moreover, only six attributes were exclusively used for females in contrast to 59 for males. Female attributes were passive (e.g., modest, noble, dear, etc.) while those for a male were bold (brave, truthful, etc.).

In a more recent study in the Sindh province, a study found 60% of the stories to be pro-male, while 76% of all images contained a male character [85]. In the Punjab region, content analysis of primary school Urdu and English language textbooks revealed similar evidence of gender stereotypes and ‘gender apartheid’ pictures [86]. A comparative study of three South Asian countries: Pakistan, Bangladesh, and Nepal also confirms underrepresentation of women, particularly in Pakistani textbooks [87].

In Bangladesh, while textbook contents have often been manipulated by successive governments for political purposes [88], gender bias in the curriculum and learning materials remains an overlooked topic. Because of the excessive focus on creating a national identity through textbooks, issues like gender are often bypassed by the authority in charge of textbook development [87,89]. The available evidence is mostly qualitative. The findings indicate that women are given limited space, and their contribution is disregarded in the textbook [87,89,90].

In sum, our review of the existing literature spanning the period 1980–2016 altogether identified nineteen studies covering Malaysia, Indonesia, Pakistan and Bangladesh. The overall findings from these studies, as discussed above, show broadly similar patterns of gender stereotypes over time and across countries. Moreover, the evidence discussed for developing countries also show high gender stereotypes regardless of whether they are Muslim or non-Muslim, Arab or non-Arab, Asian or African. Findings based on more recent analysis in these countries do not suggest that exclusion and improper representation is decreasing. Nonetheless, it is difficult to conclude whether the observed pattern is systematic across countries. Most of the studies reviewed in this section suffer from several methodological limitations which limit the scope for direct comparisons.

First, they employ a narrow selection of textbooks (regarding the total number and types) and may not be representative of the true extent of gender bias. Second, most studies rely on a very small number of categories. Third, some studies only examine pictures (e.g., Ena [83] in Indonesia) while others focus on texts. In general, there appears to be a trade-off between content and textbook coverage in the literature–studies covering more textbooks restrict the analysis to fewer content-specific indicators. For instance, Jasmani et al. use six textbooks but only one indicator [84]. Apparently, there is no consensus among scholars regarding the number of textbooks and categories for constructing the quantitative indicators. The majority of the studies employed 4 to 5 indicators, on average, while the number of textbooks used range from 1 to 194. Therefore, in this study, we address these methodological limitations not only by using a much larger sample of indicators/categories, but also by examining the robustness of our findings by varying the textbook sample for one study country by grade, province, and subject. To our knowledge, this is the first study looking at developing countries to perform such a sensitivity analysis formally.

Lastly, the literature also lacks consensus on what constitutes ‘misrepresentation’. According to policymakers in some countries, textbooks should represent the social reality in a country [84]. A higher presence of women in lower socioeconomic roles and characters is therefore an authentic portrayal of women even though they may reinforce gender stereotypes. Depicting women as leaders and decision makers in society and men as caregivers may constitute a misrepresentation. Others, however, maintain that the role of the textbook should be emancipatory and present progressive and affirmative images of women [52,91]. Women’s representation in textbooks should reflect their dynamic positions, multiple roles and identities; they may be mothers and caregivers but also income earners and professionals [92,93]. In this paper, our approach is the latter and we evaluate the quality of representation in text and images in terms of proportionate gender presence in prestigious roles and activities.

## Research design, the sample, and data

Our primary study population of interest is secondary school English language textbooks used in the ninth grade in the academic year of 2015 [94–97]. Sample textbooks are described in Table 2. There are two reasons for restricting the analysis to English language textbooks. Firstly, as this is a cross-country study, only English language textbooks can be analyzed comparatively without any language barriers. Secondly, English language textbooks have been widely used in gender stereotype research and other socio-cultural content analysis studies for both single country analysis [59–61,63–65,70,72,73,80,82,86,98] as well as cross country comparisons [99].

**Table 2:** Description of the sample textbooks^#^.

	Textbook name	Authors	Grade	Year of publication/re-print
Malaysia^€^	English Form 4	Tan Phaitk Lee and Angelina Ng Kim Leng*	9	2011
Indonesia^€^	English in Focus	Artono Wardiman, Masduki B. Jahur and M. Sukirman Djusma	9	2008
Pakistan (Punjab)^€^	English 9	Mrs Sahida Rasul* and Mrs Sabiha Salim*	9	2016
Bangladesh^€^	English for Today	Raihana Shams*, Md. Zulfeqar Haider, Goutam Roy, Surajit Roy Majumder, Md Abdur Razzaque and Naina Shahzadi*	9–10	2012
KPK board^**¥**^	Textbook of English	Ruhi Zaka Malik*	9	2011
English	English 4	Iftikhar Selim and Nabila Gull*	4	2016
English	English 6	Mumtaz Ahmad, Munazza Tazammal, Tayyaba Sadia*, Mirza Ghulam Muhammad Baig	6	2016
English	English 7	Qazi Sajjad ahmad and Rana Ahmad Shaheed	7	2008
English	English 8	Rafiq Mahmood, B. A Chishty, Z.I Farooqi, Muhammad Aslam and Mrs. Sabiha Salim*	8	2012
English	English 10	Sabia Kiyani*	10	2013
Chemistry	Chemistry	Dr. Jalil Tariq and Dr. Irshad Ahmed	9	2016
Physics	Physics	Prof. Tahir Hasan and Prof. Muhammad Naeem Anwar	9	2016
Biology	Biology	Unnamed	9	2016
Mathematics	Mathematics	Dr. Karamat H. Dar and Prof. Irfan ul Haq	9	2016

Note:

^#^This table describes textbooks used in the main analysis.

* indicates female authors

^€^ indicates English language textbook for grade 9.

^**¥**^ indicates Pakistani textbooks.

We use ten additional textbooks from KPK province of Pakistan for sensitivity analysis which we further describe in section 5.3. KPK = Khyeber Pakhtunkhwa.

The justification for focusing on secondary school textbooks is twofold. First, some studies used secondary level textbooks for analyzing gender stereotypes [60,61,63,64,70–72,78,80,82,98,99]. Second, this is the final stage for students after which they decide to either further their academic career, or enter the job market (primarily boys), or get married off (primarily girls).

Our analysis of gender stereotypes in textbooks is based on two broader frameworks, i.e exclusion and the quality of representation. ‘Exclusion’ refers to the lack of presence of a particular gender, while poor ‘quality of representation’ refers to the incorrect or biased portrayal of one gender over the other (see Table 3). According to OHCHR [58], gender stereotypes can arise in either form and are defined as:

“*…a generalized view or preconception about attributes or characteristics that are or ought to be possessed by*, *or the roles that are or should be performed by women and men*. *A gender stereotype is harmful when it limits women’s and men’s capacity to develop their personal abilities*, *pursue their professional careers and make choices about their lives and life plans*. *Harmful stereotypes can be both hostile/negative (e*.*g*., *women are irrational) or seemingly benign (e*.*g*., *women are nurturing)*.”

**Table 3:** Breakdown of categories for content analysis.

**A. Indicators of exclusion**	
** Text content: non-pictorial**	**Visual representation—Picture**
**Words**	
Names (range) ^1^	Total number of pictures^6^
Names (total) ^1^	Centeredness (of the picture)
Nouns	Indoor activity (in the picture)
Pronouns	Outdoor activity (in the picture)
Attributes (range)	
Attributes (total)	
Social role^2^	
Professional occupation	
Activities (range)	
**Sentences**	
Firstness	
Dialogue	
**Stories**	
Author ^3^	
Leading character ^4^	
Centeredness	
Total characters^5^	
**B. Indicators of the quality presentation**	
Domestic roles	
Term used to address females	
Professional roles	

Notes

^1^ “Range” refers to the number of distinct variables (such as names and occupations) and “Total” refers to the number of times the variable was repeated in the textbook. For instance, if the same name is mentioned five times on a page, this corresponds to a one in the ‘Names (range)’ category and a five in the ‘Names (total)’ category.

^2^ The roles played by female-male characters outside the domestic boundary (e.g. teaching, driving car etc.).

^3^ This includes author of the whole textbook as well as stories and poems within the textbook.

^4^ Number of male-female characters/persons in the leading role; This includes author of the whole textbook as well as stories and poems within the textbook

^5^ Number of male-female characters/persons in any role.

^6^ Total number of pictures with at least one male-female character.

However, the sum of the other three pictorial indicators may not add up to the total number of picture. Because, a picture with a female-male character might not necessarily show any of the other three characteristics (e.g., centeredness, indoor and outdoor).

Keeping the above definition in mind, and building on the literature discussed earlier, 21 sub-categories were identified. Our qualitative, albeit descriptive, analysis is primarily carried out using four elements of the textbook content. These are: words (e.g. names, nouns, pronouns, attributes, roles etc.), sentences (e.g. dialogues between two males vs. two females, firstness or order of mention in sentences), stories (e.g. centeredness of the story, leading characters) and pictures (e.g. the individual and their activities) as different units of analysis. We follow the four Cs of Cohen—coding, categorizing (creating meaningful categories where suitable units of analysis are used), comparing and concluding [67]. A total of 713 pages from sample textbooks were manually analyzed based on the 21 identified categories in a Microsoft excel spreadsheet.

To ensure the reliability of the content analysis, we followed two strategies. First, a small subset of textbook pages (20%) was initially analyzed to check the appropriateness of categorization as suggested by Weber [69] and Cohen [67]. Second, following Milne and Adler [100], overall findings were cross-checked after a few weeks of the primary content analysis to ensure inter-coder reliability.

Since our study of gender stereotypes is centered upon two broad frameworks, exclusion and the quality of representation, the 21 categories are organized to fit these two broad frameworks. Data on each of the 713 pages of the four textbooks are analyzed according to these categories and inputted into the excel spreadsheet using simple codes. After the frequency and range of categories are identified, data analysis was done at the individual level (i.e., gender wise analysis) as well as at the aggregate level (i.e., cross-country analysis). The data was analyzed in order to identify patterns and differences between genders and countries.

It should be noted that, similar to previous studies, we examined school textbooks by restricting the analysis to a single-subject textbook, as well as from a single grade. However, as discussed in section 3, this is less than ideal since the findings, particularly country rankings, can be sensitive to sample composition. Moreover, in countries governed by a federal system, textbook content can vary by location. To address these concerns, we examined additional textbooks to verify whether our results change depending on the textbook type. We do so by looking at five additional English textbooks, from both primary and secondary school levels, as well as four subject-specific books used in grade 9 in the Punjab province of Pakistan. This sensitivity analysis is, however, based on pictorial indicators only. To assess whether the results are specific to the Punjab province, we repeated the full content analysis (text as well as picture) using the grade 9 English textbook from KPK, one of the poorest provinces of Pakistan (see Table 2 for details).

## Main findings

### Gender visibility: Exclusion vs. inclusion

Exclusion or under-representation of one gender in the textbook is one form of gender stereotype and discrimination. Table 4 presents our findings for each category using both text and pictures. The figures in the table show the percentage of female presence for each indicator and the figure in parenthesis indicates the total number of the respective item in the textbook.

**Table 4:** Female exclusion in text and illustrations by country.

	Malaysia	Indonesia	Pakistan	Bangladesh	4-country
			(Punjab)		average
**Text content: non-pictorial**					
**Words**					
Names–total	50.4	42.9	13.6	36.6	35.9
	(781)	(407)	(168)	(713)	(2069)
Names–range	44.7	47.8	16.6	39.4	37.1
	(447)	(232)	(72)	(274)	(1025)
Nouns	46.7	33.0	46.9	33.9	40.1
	(622)	(224)	(307)	(513)	(1666)
Pronouns	46.5	40.3	21.5	35.2	35.9
	(1682)	(625)	(288)	(1509)	(4104)
Attributes–total	54.5	33.3	19.0	47.1	38.5
	(455)	(147)	(21)	(242)	(865)
Attributes–range	44.1	31.0	25.0	30.1	32.5
	(147)	(54)	(12)	(136)	(349)
Social role	44.1	44.0	7.6	29.1	31.2
	(215)	(59)	(26)	(254)	(554)
Professional occupation^b^	34.5	15.3	64.6	23.7	34.5
	(55)	(26)	(82)	(80)	(243)
Activities–range	41.7	48.3	20.0	46.4	39.1
	(75)	(31)	(5)	(70)	(181)
**Sentences**					
Firstness	20.0	27.7	0.0	25.7	18.4
	(45)	(18)	(07)	(35)	(105)
Dialogue	68.7 (16)	68.1 (22)	-	62.5 (08)	66.4 (46)
**Stories**					
Author	60.7	36.3	50.0	43.7	47.7
	(28)	(22)	(6)	(16)	(72)
Leading character	43.2	48.8	10.0	42.5	36.1
	(238)	(174)	(22)	(155)	(589)
Centeredness	56.4	45.0	26.6	37.1	41.3
	(39)	(20)	(15)	(35)	(109)
Total characters	47.4	45.8	16.4	36.4	36.5
	(674)	(303)	(97)	(351)	(1425)
**Sub-total (non-pictorial)**	46.9	40.5	24.4	37.9	38.0
**Visual representation—Pictures**^**a**^					
Total number of pictures	40.9	55.9	-	28.5	41.8
	(376)	(168)		(140)	(684)
Centeredness (of the picture)	34.8	54.5	-	31.1	40.1
	(152)	(44)		(45)	(241)
Indoor activity (in the picture)	27.2	66.6	-	54.5	49.4
	(33)	(6)		(11)	(50)
Outdoor activity (in the picture)	38.0	55.0	-	26.9	40.0
	(168)	(80)		(63)	(311)
Sub-total (pictures)	35.2	58.0	-	35.2	42.8
Grand Total % (text & pictures)	44.4	44.1	24.4	37.3	40.4

Note

(a) The Pakistani (Punjab) sample textbook contained no human pictures/figures and no single-sex dialogues.

(b) The unusually high female share in the professional occupation category in the Pakistani textbook is because of ‘ghettoization’–most of the occupations were mentioned on two pages corresponding to a chapter entitled “Women Arise.”

In the nine word-related sub-categories, the average female share is 36.08% (aggregating the four-country average). Regarding the sentence-related sub-category, average female exclusion is 42.4% in firstness and dialogue indicators. In the sub-category relating to stories, we found female presence to be 40.4% in the four categories. Finally, the visual representation sub-categories show the female share to be 42.8%. Findings based on 19 indicators reported in Table 4 show that female presence is lower (40.4%) than male presence.

However, there is important variation across countries regarding female exclusion. The sum of 19 categories shows a nearly balanced percentage share between males and females in Malaysian (44.4) and Indonesian (44.1) textbooks. In contrast, we find high female exclusion in Pakistani (24.4) and Bangladeshi (37.3) textbooks. This finding is consistent with female share in non-pictorial indicators. However in pictorial indicators, the Indonesian textbook has a very high female share (58.0%), while the Malaysian and Bangladeshi textbooks have a female share of 35.2%. There are no images with human figures in the Pakistani textbook (from the Punjab board). Also, even though there is apparent gender parity in the Malaysian and Indonesian textbooks, we still find considerable gender disparity when individual indicators are considered. For instance, high presence of male characters is evident in categories like ‘Firstness’, ‘Professional occupation’ in the Malaysian textbook and ‘Nouns’, ‘Attributes (range)’, ‘Attributes (range)’, ‘Professional Occupation’, ‘Firstness’ and ‘Author’ in the Indonesian textbook (see Table 4).

To better understand exclusion in the context of previous studies, Table 5 below summarizes the findings of female-male visibility in previous studies of five developed non-Muslim majority countries and five developing Muslim majority countries. The findings of our study resemble all the recent studies conducted in developing countries as well as the findings from studies and textbooks used in developed countries two to three decades ago. All these studies identified the share of male presence between 65–75 percent and the share of female presence between 25–35 percent in the textbooks.

**Table 5:** Overall representation of women (in percentage), sample composition and indicators used in selected published studies^a^^,^^b^.

Studies		Country	Number of Sample books	Number of indicators used	Type of content analyzed			Female presence (%)
					Text	Picture	Both	
**Our study**		Malaysia	1	21			☑	44.4
		Indonesia	1	21			☑	44.1
		Pakistan	1	21			☑	24.4
		Bangladesh	1	21			☑	37.3
**Existing studies**								
**Developing Muslim countries**								
	Jasmani et al. [80]	Malaysia	6	5	☑	×		34
	Ena [82]	Indonesia	8	2	×	☑		37
	Chandrawati [83]	Indonesia	1	-	☑	×		23
	Mirza [84]	Pakistan	194	5			☑	20–25
	Jabeen and Ilyas [85]	Pakistan	10	7			☑	31
	Kaya [101]	Turkey	30	2			☑	37
	Hall [61]	Iran	2	2			☑	31
	Jaber MA [102]	Jordan	38	9			☑	20
	**Developed country (non-Muslim)**							
	Gouvias and Alexopoulos [103]	Greece	5	16	☑	×		28
	Farree and Hall [104]	USA	33	1	×	☑		36
	Cooke-Swayer [105]	Canada	7	3			☑	18
	Mineshima [63]	Japan	1	8			☑	50
	Lee and Collins /old books [64]	Hong Kong	10	7			☑	37
	Lee and Collins /new books [64]	Hong Kong	10	7			☑	51
	Blankenship [106]	USA	4	4			☑	46

Notes

(a) This table lists published studies that report quantitative findings of gender stereotypes. Existing studies using Bangladeshi textbooks are not mentioned in this table since they are all based on qualitative evidence.

(b) A handful of studies listed here only measure the extent of gender stereotypes in ratios or frequencies.

We have converted them to percentages for easy comparison.

However, the country-specific findings of this study reveal surprising facts about exclusion. It shows that the degree of exclusion in the Malaysian and Indonesian textbooks is quite different from the degree of exclusion in the Pakistani and Bangladeshi textbooks. The former two countries resemble the findings of recent studies in developed countries showing a more balanced gender representation. On the other hand, in the Pakistani and Bangladeshi textbooks, the degree of exclusion also varies but resembles findings of developing countries in general. While the Bangladeshi textbook almost resembles the overall finding of this study, the Pakistani textbook represents a much higher female exclusion, which is consistent with findings from recent studies using Pakistani textbooks.

### Improper representation

In addition to the under-representation of one gender in textbooks, gender stereotyped representations or false portrayal of one gender is another form of gender stereotyping. In this section, we discuss the ‘quality of gender representation’, based on four categories: ‘terms used to address females’, ‘domestic roles’, ‘professional roles’, and ‘attributes’ used for female and male characters (see Tables 6–9). In contrast to the categories reported in Table 4, which are primarily quantitative measures of female exclusion, these four categories capture both the quantitative and qualitative aspects of gender representation.

**Table 6:** Female presence in domestic and social roles.

Indicator	Malaysia	Indonesia	Pakistan	Bangladesh	Average across countries
Social role	44	44	8	29.1	31.2
	(215)	(59)	(26)	(254)	
Domestic role	85	63	100	73	80.3
	(23)	(8)	(2)	(15)	

**Table 7:** Terms used when addressing female characters.

	Malaysia		Indonesia		Pakistan		Bangladesh	
	Miss	Mrs.	Miss	Mrs.	Miss	Mrs.	Miss	Mrs.
Frequency	28	8	4	12	0	0	11	0
%	78	22	25	75	0	0	100	0

Note: We did not find any examples of any of the terms in the Pakistani textbook.

**Table 8:** Most stated professional roles/occupations by gender.

	Malaysia	Indonesia	Pakistan	Bangladesh
**Female professions**	Teacher (2)	Singer (3)	Nurse (4)	Teacher (13)
	Maid (2)	Dancer (2)	Midwife (4)	Lawyer (3)
			Poetess (3)	TV anchor (3)
			Author (2)	Social Scientist (2)
				Queen (2)
**Male professions**	Doctor (8)	King (4)	Poet (11)	Custom officer (9)
	Poet (5)	Musician (4)	Writer (10)	Smuggler (6)
	Manager (4)	Firefighter (2)	President (6)	Sprinter (4)
	Drover (4)	Postman (2)	Author (5)	Poet (4)
	Actor (2)	Singer (2)	King (4)	President (4)

Note: Only roles mentioned more than once are taken into account.

**Table 9:** Gender wise attributes at each indicator of FFM (High and Low).

	Malaysia		Indonesia		Pakistan		Bangladesh	
	Female	Male	Female	Male	Female	Male	Female	Male
Extroverted and active personality								
High E	67	33	0	100	0	100	13	87
High O	40	60	20	80	17	83	16	84
High C	33	67	0	0	100	0	29	71
Low N	0	0	0	0	0	100	0	100
Low A	50	50	50	50	17	83	0	100
Ratio points	1	2	0	2	1	4	0	5
Introverted and passive personality								
Low E	20	80	0	0	0	100	0	100
Low O	100	0	0	100	0	0	0	100
Low C	50	50	0	0	0	0	100	0
High N	57	43	0	0	0	100	100	0
High A	63	37	0	100	23	77	33	67
Ratio points	3	1	0	2	0	3	2	3

Note: All figures in %.

#### Representation in domestic roles

The high percentage of female characters in domestic roles is one of the common forms of gender-stereotyped representation in textbooks [64,84]. We find that females are presented in domestic roles four times more than their male counterparts (see Table 6). The four-country average female representation in different social roles is 31.2%. Even for the Malaysian textbook, which have a fairly balanced female aggregate presence using the 19 indicators (i.e. Table 2), we find a high female representation in the domestic role category (with a share of 85%). Meanwhile, in the Pakistani textbook, no male character is depicted in a domestic role.

#### Terms used when addressing female characters

When a female is addressed as ‘Mrs.’, it identifies her in terms of her male counterpart. On the other hand, when someone is addressed as ‘Miss or Ms.’, then it refers only to her identity. The use of ‘Mrs.’ or ‘Miss/Ms.’ is thus an important category in linguistic sexism and has been used in previous content analysis studies [64]. In our study, both Malaysian and Bangladeshi textbooks show more frequent use of ‘Miss’ in addressing female characters, while in Indonesian textbook, most female characters are referred as ‘Mrs.’ (see Table 7). This shows a promising shift in the use of terms when addressing female characters, moving from focusing on their marital identity to their independent identity.

### Representation in professional roles or occupations

Table 8 below shows the most stated professions for both female and male characters. ‘Teacher’ is the most stated profession for females in Malaysia and Bangladesh. The overall findings show that professions attached to female characters are traditional, and lower in prestige and income. This finding is in line with previous studies using Pakistani, German and other textbooks [60,65,78,107,108]. Moreover, surprisingly in the Bangladeshi textbook, the professional roles are more prestigious and demanding than in any of the other textbooks (such as a lawyer, social scientist or even a TV anchor).

### Representation in personality traits

In the textbooks, women are often found to be portrayed as weak, victimized, passive and subordinate [60,70,84,99,109]. On the other hand, the image of male characters reflects quite the opposite personality; as they are portrayed as bold, brave and active agents in society. In this study, we used the five factor model [110] to identify the personality traits of female and male characters in the textbooks (see Table 9). The five factor model [110] is a popular method used in studies dealing with personality traits; it identifies five different traits: extraversion, openness, conscientiousness, neuroticism and agreeableness [111].

This study uses a list of adjectives developed by John [112] for each of the five personality traits. The adjectives are divided into two groups: high and low. Using this list, an individual is given either a high or a low score for each personality trait. A high score in extraversion (E), openness (O) and conscientiousness (C) is quite the opposite of a high score in neuroticism (N) and agreeableness (A). For instance, having a low score in the former three is the same as being introverted, closed, and having a lack of direction [111,113]. Whereas having a low score in the latter two is tantamount to being emotionally stable and analytical.

Table 9 shows female-male percentage share at each of the personality traits categories. From a total of 349 attributes range (see Table 4), we selected only those (n = 132) that correspond with the list of John [112]. Of the 132 selected attributes, only 39 attributes were found for women (29.5%) i.e. male figures have higher representation. Therefore, the direct reading of the table can be misleading, if one intends to compare gender-wise share at each of the five low and high traits. For instance, having 0% ‘Low E’ for females does not mean that they are less likely to be introvert. Rather, we have 0% ‘Low E’ due to the fact that they have exclusion problem in general. Therefore, a better way to identify male-female personality trait is through extrovert-introvert ratio of male and female for each country for each of the five traits (i.e. EOCNA).

We calculated the extrovert-introvert ratio by giving 1 point to males or females if their share is more than 50% in any of the particular categories of EOCNA. Thus, an absolute extroversion will show extrovert-introvert ratio would entail a result of 5:0 and 0:5 for absolute introversion. Our result shows that for males, the extrovert-introvert ratio is 2:1, 2:2, 4:3 and 5:3 for Malaysia, Indonesia, Pakistan and Bangladesh and ratio of 1:3, 0:0, 1:0 and 0:2 for females respectively. This means, except for Indonesia, the other three countries have high male share in favor of extroverted traits and introverted ones. Similarly, for females, only Pakistani textbook favors extroverted traits (that too is due to exclusion problem). On the other hand, both Malaysian and Bangladeshi textbooks have high female share in introverted traits than extroverted ones. In our sample, Malaysia is the only country where we find equal male-female attributes range for testing the attributes (the other three has exclusion problem). Even for Malaysia our result show males favoring extroverted personality traits while females as introvert and passive, a finding that is consistent with past studies [27,80,84,99,109].

Besides, this demarcation is also visible how female and male characters are presented in the textbooks. Some of the common characters attributed to men include: *disciplined*, *responsible*, *sensible*, *visionary*, *legendary* etc., whereas for females the attributes include: *messy*, *depressed*, *kind*, *compassionate* etc. Some of the practical examples from the textbooks for females are: a) “I am a sixteen year old girl. I am depressed.” (p.168 in Malaysian textbook); b) “Tisha’s room is always messy” (p.8 in Bangladeshi textbook); c) “the world salutes her for her love and compassion for humanity” taking about Mother Teresa (p. 118 in Bangladeshi textbook); d) “Nature, a mother, is kind to all” (p. 122 in Pakistani textbook) and for males examples include: a) Jamil, a sensible character who is lauded for avoiding overcrowded boat and saving his life. (p. 49 in Bangladeshi textbook); b) “Jobs was a visionary” (p. 121 in Bangladeshi textbook); c) “They came together to oppose this powerful giant by using his stupidity” talking about a character named Kbo Lwo in Bali (p. 91 in Indonesian textbook).

## Sensitivity analysis

One criticism of the existing quantitative analyses of textbook content is that they rely on very specific samples and, therefore, it is difficult to generalize the results. Our analysis can be criticized on the same grounds, as we have only used grade 9 English textbooks from each of the study countries. In this section, we, therefore, assess the sensitivity of our findings by using additional textbooks from one of our study countries. Since Pakistan is ranked behind other sample countries in terms of…, we conduct the sensitivity analysis using other Pakistani textbooks. We do this to ascertain whether the poor ranking is as a result of the textbook we chose, or due to the absence of other variables causing heterogeneity in our data.

First, we repeat the analysis of the picture contents of English textbooks used in different grades in the Punjab province (see Table 10). The grade 9 sample textbook has no pictures, while all the books used in grades 4 to 8 and 10 contain pictures. A total of 575 pages from five textbooks were analyzed using pictorial indicators. The percentages in Table 9 show that in all secondary school textbooks, Pakistan is behind the other sample countries regarding female presence (see Table 4). This is true in all domains, i.e., the total number of pictures, the centeredness of the picture and indoor and outdoor activities. The percentage of pictures with females ranges from 11 to 25 percent (considering all grades). Only the grade 4 textbook appears to have a balanced representation of males and females.

**Table 10:** Grade-wise analysis of female presence in English language textbooks in Pakistan (the Punjab province only).

	Grade 4	Grade 6	Grade 7	Grade 8	Grade 10
Total number of pictures	52	25	11	17	19
	(54)	(131)	(9)	(12)	(16)
Centeredness (of the picture)	56	19	11	14	13
	(9)	(47)	(9)	(7)	(114)
Indoor activity (in the picture)	67	50	0	0	50
	(9)	(6)	(6)	(0)	(2)
Outdoor activity (in the picture)	45	19	0	14	17
	(22)	(41)	(2)	(7)	(6)
**Total %**	**55**	**28**	**6**	**11**	**25**

Second, we assess whether the poor ranking of Pakistan is because we restricted our analysis to English language textbooks only. We repeat the analysis of the Pakistan textbooks (from the Punjab province) using grade 9 Chemistry, Mathematics, Science, and Biology textbooks. In total, we examined pictorial contents from 945 pages for this analysis. As can be seen from Table 11, grade 9 textbooks from the Punjab province, in general, contain very few pictures. In the Physics book, we identified 23 pictures (containing a human character) of which only 17% contained a female figure. Chemistry and Biology books contained as many as 58 and 21 pictures with human figures respectively, and all of them were male.

**Table 11:** Subject-wise analysis of female presence in grade 9 textbooks in Pakistan (the Punjab province only).

	Chemistry	Math	Physics	Biology
Total number of pictures	0	0	26	0
	(58)	(1)	(23)	(21)
Centeredness (of the picture)	0	0	20	0
	(57)	(1)	(15)	(12)
Indoor activity (in the picture)	0	0	0	0
	(0)	(0)	(0)	(0)
Outdoor activity (in the picture)	0	0	21	0
	(48)	(0)	(78)	(0)
**Total %**	**0**	**0**	**17**	**0**

Lastly, we repeated the same analysis as in Table 4 using a grade 9 English textbook from KPK, a different province in Pakistan (see Table 12). In 9 out of the 19 non-picture related categories, the female share is lower in the KPK textbook as compared to the Punjab textbook. While KPK English textbook for grade 9 contains many pictures (when that for Punjab contains none), the overall female share in pictures is very small (3%) and far below the respective figures for other study country samples (as shown in Table 4). Consequently, the overall result (picture and non-picture items combined) for the KPK textbook is 14% which is even lower than that reported earlier for the Pakistani textbook in Table 4.

**Table 12:** Province-wise results of female presence in the English language textbooks in Pakistan (grade 9).

	KPK Board	Punjab Board
**Text context: non-pictorial**		
**Words**		
Names (range)	12	17
	(147)	(72)
Names (total)	6	14
	(300)	(168)
Nouns	27	47
	(369)	(47)
Pronouns	20	22
	(801)	(288)
Attributes (total)	29	19
	(157)	(21)
Attributes (range)	22	25
	(113)	(12)
Social role	16	8
	(141)	(26)
Professional occupation	19	65
	(48)	(82)
Activities (range)	25	20
	(60)	(5)
**Sentences**		
Firstness	0	0
	(50)	(7)
Dialogue	-	-
**Stories**		
Author	21	50
	(78)	(6)
Leading character	14	9
	(79)	(22)
Centeredness	15	27
	(50)	(15)
Total characters	26	16
	(296)	(97)
**Sub-total % (non-pictorial)**	17	24
**Visual representation—Pictures**		
Total number of pictures	3	-
	(124)	
Centeredness (of the picture)	9	-
	(22)	
Indoor activity (in the picture)	0	-
	(2)	
Outdoor activity (in the picture)	0	-
	(78)	
**Sub-total % (pictures)**	**3**	**-**
**Grand Total % (text & pictures)**	**14**	**24**

In sum, the analysis of textbooks from a different province (KPK) as well as those relating to different subjects and grades in the Punjab province confirm that at least in the context of Pakistan, findings based on our main analysis are not driven by the textbook subject or grade. Irrespective of what textbook we used from Pakistan, it lagged behind the other study countries regarding female presence in textbooks.

## Conclusion

We found a high degree of gender stereotypes in the form of ‘exclusion’ and ‘the quality of representation’ in all the sample textbooks. Moreover, female characters were mostly associated with traditional and low wage occupations as well as more passive personality traits. The extent of stereotypes found in the textbooks, however, varies across countries. The Malaysian and Indonesian textbooks have a more egalitarian representation of females than their South Asian counterparts, Pakistan and Bangladesh. On the other hand, compared to the other three countries, women in Bangladeshi textbooks are described in a wider range of professional roles such as teachers and lawyers. Overall, the contents of the Pakistani textbook show the highest percentage of stereotypes (regarding exclusion), while the lowest was found in the Malaysian textbook. The Pakistani textbook consistently ranks below the Malaysian, Indonesian, and Bangladeshi in almost all gender indicators. Using province, subject and grade specific textbooks, we further show that the poor ranking of the Pakistani textbook, in comparison to the other study countries is not as a result of the specific textbook used in our primary analysis. Among other things, while textbooks in Indonesia and Malaysia have higher gender balance when compared to Pakistan, our analysis also highlights areas where there is room for further improvement in textbook gender content (regarding quality of representation) in these two countries. Nonetheless, the observed regional differences are consistent with the relatively higher economic status women in Southeast Asian countries enjoy in comparison to other developing countries [115,116] and in line with evidence presented in earlier studies using textbook content analysis [87].

Our findings have important implications for gender and education policies in developing countries. Public interventions in South Asia that focus on women’s development have been preoccupied with employment and income-generating schemes [117]. This approach implicitly assumes that the education system will empower women by preparing them for the job market. Our results highlight the need to go beyond the current policy focus on improving access to education among girls. In that sense, our findings add to the recent research that warns about the limitations of fostering gender equity exclusively through school-based initiatives [118,119, 122]. In this regard, the Global Monitoring Report 2015 rightly stressed the need to revise textbook content and restore gender balance as well as encourage children to question gender stereotypes in the society [120]. However, not all governments have been equally successful in addressing the problem. In the case of Pakistan, the 2001–2015 Education for All (EFA) action plan also acknowledged the need to free textbooks of gender bias. Despite this policy initiative and clear evidence of gender bias in learning materials documented in academic research conducted in the 1990s and 2000s, we found evidence of gender stereotypes in Pakistani school textbooks across grades, subjects, and provinces. The lack of change in textbook content in Pakistan partly reflects the fact that chairmen and directors of textbook boards in the country believe that gender portrayals of the textbooks should be in agreement with the status quo [84]. This suggests that simply re-prioritizing the elimination of stereotypes in school textbooks and classroom practices in policy documents may not be enough to attain the SDGs of gender equality by 2030. Changing the mindset of policymakers remains a key challenge. A participatory approach that ensures a wider consultation of teachers, authors, and reviewers of both genders in curriculum and textbook review as well as development is critical [93]. Such consultation processes should also include comprehensive expert reviews of textbook gender contents such as this one.

Lastly, while the extant literature primarily documenting gender bias in curriculum materials is growing, why countries differ in terms of textbook quality is unclear. One popular explanation is the male dominated textbook development process [93], though it is difficult to statistically validate this hypothesis based on a small sample of textbooks. Political factors can also influence curriculum development, particularly in countries where the delivery of education is decentralized to the state level. Moreover, very few studies have attempted to evaluate the impact of programs which make textbooks gender-sensitive [121]. Evidence suggests considerable variations in support for gender stereotypes among students across schools that differ in terms of curriculum content [122]. Follow-up studies focusing on the causes of cross-country variation in gender contents as well as evaluation of existing initiatives to remove gender bias in textbook content will be informative. Equally, while our analysis confirms that textbooks disseminate a hidden curriculum among students, we did not empirically test whether actual student attitudes systematically vary owing to the textbooks they used in school. This is left for future research.
